# Longitudinal Surface Water Viral Surveillance Following the 2024 Extreme Flood: One-Year Monitoring in Porto Alegre, Brazil

**DOI:** 10.1007/s12560-026-09713-2

**Published:** 2026-10-26

**Authors:** Thales De Lima Bermann, Fernanda Mückler-Pereira, Bruno Aschidamini Prandi, Nicóle Verardo De Mattos, Vítor Felippi Tessele, Luis Gustavo Dos Santos Da Silva, Nicole Vieira Stone, Raíssa Gasparetto, Bruna Simone Paredes-Galarza, Martha Trindade Oliveira, Louidi Lauer Albornoz, Lucas Bohnenberger, Salatiel Wohlmuth da Silva, Ana Cláudia Franco

**Affiliations:** 1 Virology Laboratory, Microbiology, Immunology and Parasitology Department, Institute of Basic Health Sciences, Federal University of Rio Grande do Sul (UFRGS), Ramiro Barcelos St. 2600, Porto Alegre, Brazil; 2 Genetics Department, Institute of Biosciences, Post-Graduate Program in Genetics and Molecular Biology (PPGBM), Federal University of Rio Grande do Sul, Porto Alegre, Brazil; 3 Center of Environmental Sanitation Studies (NESA) and Post-Graduate Program in Water Resources and Environmental Sanitation (PPG-IPH), Porto Alegre, Brazil

**Keywords:** Extreme flood events, Environmental viral surveillance, Enteric viruses, Norovirus, Hepatitis A, Hepatitis E, SARS-CoV-2

## Abstract

Extreme flood events can overwhelm sanitation infrastructure and drive the widespread dispersal of viral pathogens into aquatic environments. In May 2024, Porto Alegre (southern Brazil) experienced an unprecedented flood that caused record-breaking overflow of the Guaíba hydrological system and prolonged urban inundation. Here we report a one-year longitudinal environmental viral surveillance study, comprising seven campaigns between May 2024 and April 2025. Surface waters were analyzed by RT-qPCR for SARS-CoV-2, norovirus genogroups I and II, hepatitis A virus (HAV) and hepatitis E virus (HEV). All five targets were detected simultaneously during the flood peak, reflecting widespread contamination possibly driven by sewage overflow and multi-site infrastructure failure. Thereafter, the number of targets detected per campaign was lower and fluctuating (two to four), tracing a transition from an initial multi-pathogen contamination pulse observed during the flood peak to persistent detection of viral genomic material in surface waters. SARS-CoV-2 was detected in every campaign, in a minority of sites in all campaigns except the last (C7, 14/22, 63.6%), where both detection frequency and viral load increased significantly (*p* < 0.001 for both; median Ct 29.7 versus 36.8). HEV was detected only at the flood peak; norovirus GII was detected intermittently up to C6, without a significant temporal pattern (*p* = 0.13); and norovirus GI was absent between C2 and C5, reappearing in C6 and C7. HAV detection increased significantly over the monitoring period (Cochran–Armitage *p* < 0.0005), temporally coincident with an officially declared hepatitis A outbreak. These findings demonstrate the value of longitudinal, multi-target environmental surveillance for capturing both the acute virological impact of extreme flooding and the persistent contamination dynamics that follow, pointing to chronic vulnerabilities in urban wastewater infrastructure that outlast the emergency.

**Supplementary Information:**

The online version contains supplementary material available at https://doi.org/10.1007/s12560-026-09713-2.

## Introduction

In southern Brazil, the 2024 flood affecting Porto Alegre and its metropolitan region represented a striking example of an extreme climate-related disaster intersecting with deep-seated sanitation vulnerabilities. Porto Alegre, the capital of Rio Grande do Sul State, spans 496 km^2^ with a population of approximately 1.33 million residents across 94 administrative neighborhoods (IBGE, 2023; PMPA, 2024). Between late April and early May 2024, the most intense large-scale precipitation event ever recorded in Brazil drove water levels in the Guaíba system to 5.35 m, surpassing the previous historical 1941 flood record by nearly a meter (Collischonn et al., 2025). The disaster directly inundated 46 neighborhoods, predominantly high-density urban zones across the Northern, Central and Southern sectors, displacing over 200,000 residents and causing up to 180 casualties. The crisis exposed the acute fragility of the city’s water and sanitation infrastructure, which lacked contingency operating protocols for catastrophic natural disasters.

Under normal conditions, Porto Alegre’s municipal basic sanitation system covers 91.8% of the urban population with wastewater collection. However, only 73.9% of this collected sewage undergoes treatment, relying on conventional activated sludge processes (SINISA, 2024). Rural and peripheral areas lack formal sewer networks, relying on unmonitored discharges, although official data regarding the prevalence of these practices remain limited. During the flood, the municipal water supply system collapsed completely, with 5 out of 6 water treatment plants (WTPs) shut down, primarily to prevent electrical failures, exposing an estimated 1.2 million residents to water supply disruptions. Regarding residual urban waters, 24 of the 36 wastewater pumping stations (WWPS) were fully or partially submerged (Albornoz et al., 2026). Furthermore, 6 out of 10 water resource recovery facilities (WRRFs) were rendered non-operational: Sarandi, Serraria, Lami, Belém Novo, São João-Navegantes and Rubem Berta, which represents a cumulative treatment capacity of 4,782 L/s. As untreated sewage was directly discharged into floodwaters, microbial contamination escalated, with total coliform levels exceeding legal limits by up to 800-fold (804,273 MPN/100 mL) (Albornoz et al., 2026). Further geographical and hydrological descriptions of Porto Alegre, and the 2024 flood dynamics are available elsewhere (Albornoz et al., 2026; Bohnenberger et al., 2026; Collischonn et al., 2025).

Multiple technical reports and hydrometeorological assessments have underscored the unprecedented character of the event and its regional impacts, emphasizing the vital need for systematic environmental monitoring during recovery (Mantovani et al., 2025; Medeiros et al., 2025). As extreme rainfall and flooding events are projected to increase in frequency and intensity globally, their cascading impacts on urban infrastructure and public health are no longer hypothetical. Floods can overwhelm drainage and sewer systems, trigger combined sewer overflows and wastewater bypasses, and disperse human pathogens across the built environment and receiving waters. These pathways heighten human exposure via direct contact, accidental ingestion and disruption of drinking water access, factors consistently linked to elevated risks of waterborne and sanitation-related infections during and after flooding events (Acosta-España, 2024; Jagai et al., 2015).

Environmental surveillance involving surface waters and wastewater has emerged as a complementary approach to conventional clinical monitoring, capturing aggregated pathogen signals at the community level. In the context of viral pathogens, wastewater-based epidemiology (WBE) offers cost-effective, population-wide insight on infection dynamics, particularly when clinical testing is delayed, limited, or unevenly distributed. Recent syntheses highlight how multi-target viral panels used within WBE frameworks strengthen risk assessments under extreme environmental stress and infrastructure breakdown (Carmo Dos Santos et al., 2024; Chen et al., 2024; Kasprzyk-Hordern et al., 2022). Flood-impacted waters may carry a mixture of stormwater, surface runoff, and untreated wastewater, creating conditions that favor the dissemination and persistence of excreted viral pathogens. Enteric viruses, including hepatitis A virus (*Hepatovirus ahepa* - HAV) and human noroviruses (*Norovirus norwalkensee)*, exhibit remarkable environmental stability, are primarily released in feces and frequently detected in raw wastewater; WBE frameworks have repeatedly shown that norovirus signals in wastewater can track, and in some contexts precede, clinical trends (Boehm et al., 2024; Hellmér et al., 2014; Takuissu et al., 2023). Similarly, hepatitis E virus (*Paslahepevirus balayani -* HEV), a key agent of acute hepatitis with recognized waterborne transmission potential, has also been documented across diverse water matrices, including wastewater and other impacted waters, supporting environmental surveillance as a relevant component of risk assessment (Takuissu et al., 2022; WHO, 2025). SARS-CoV-2 (*Betacoronavirus pandemicum)*, although primarily a respiratory pathogen, is also shed in human excreta and has been extensively detected in wastewater, supporting its use in WBE frameworks for tracking community infection trends as a molecular marker of human waste contamination (Chen et al., 2024; Li et al., 2022).

Despite growing evidence for viral detection in wastewater and the recognized role of floods in amplifying sanitation-related transmission pathways, longitudinal datasets tracking multiple viral targets across the entire trajectory of a flood event, from the acute peak through long-term recovery, remain scarce, particularly in Latin American urban settings. Most published work captures a snapshot of contamination during or immediately after flooding, leaving open the question of how long viral signals persist, which pathogens endure, and whether what remains reflects ongoing transmission in the population rather than residual contamination from the initial disaster (Andrade et al., 2018; Phanuwan et al., 2006).

Here, we present a one-year longitudinal environmental viral surveillance study conducted in Porto Alegre following the 2024 extreme flood event. Across seven strategically spaced sampling campaigns, RT-qPCR was deployed to detect and quantify SARS-CoV-2 and to screen for norovirus genogroups I and II, hepatitis A virus (HAV), and hepatitis E virus (HEV) genomic presences in flood-impacted surface waters. We aimed to characterize viral contamination during the acute inundation phase, track its temporal evolution throughout recovery, and determine whether persistence after hydrological normalization aligns with ongoing community transmission. By evaluating the sampling sites across distinct temporal phases, from crisis through long-term hydrological recovery, this study addresses a gap directly relevant to One Health preparedness, as extreme floods reshape the interfaces linking human waste, environmental waters, infrastructure, and exposed populations, requiring integrated surveillance beyond conventional sectoral monitoring.

## Methods

### Study Area and Flood-Impacted Water Matrix

Porto Alegre is located in southern Brazil within the Guaíba hydrological system, which receives discharge from major tributaries including the Jacuí, Gravataí, Sinos and Caí rivers. The system drains a large watershed characterized by dense urbanization and industrial activities in its metropolitan region. In April–May 2024, an unprecedented flood event caused widespread overflow of the Guaíba system across the city, resulting in extensive urban inundation and the formation of persistent flooded areas that remained waterlogged for weeks to months in several neighborhoods.

In this study, the term flood-impacted surface waters refers to water bodies formed during the flooding event, consisting of varying mixtures of river water, stormwater runoff, and untreated or partially treated wastewater released through sewer overflows and infrastructure failure, and that persisted in the city for more than 30 days. These water matrices represent potential environmental reservoirs and transmission pathways for human viral pathogens following extreme hydrological disturbances.

### Longitudinal Sampling Design

We conducted seven sampling campaigns over approximately one year, spanning the acute flood period and subsequent recovery phases, as described in detail elsewhere (Bohnenberger et al., 2026). Briefly, the sampling schedule followed a predefined temporal spacing designed to capture short-, medium-, and long-term post-flood conditions. Sampling was performed on 8–10 May 2024 (Campaign 1; peak flood period with citywide overflow), followed by campaigns approximately 13 days (Campaign 2–21 May 2024), 27 days (Campaign 3–4 June 2024), 51 days (Campaign 4–28 June 2024), 112 days (Campaign 5–28 August 2024), 205 days (Campaign 6–29 November 2024), and 355 days (Campaign 7–28 April 2025) after the initial collection. Water levels across the city remained substantially elevated through Campaign 3 before gradually receding.

**Fig. 1:**
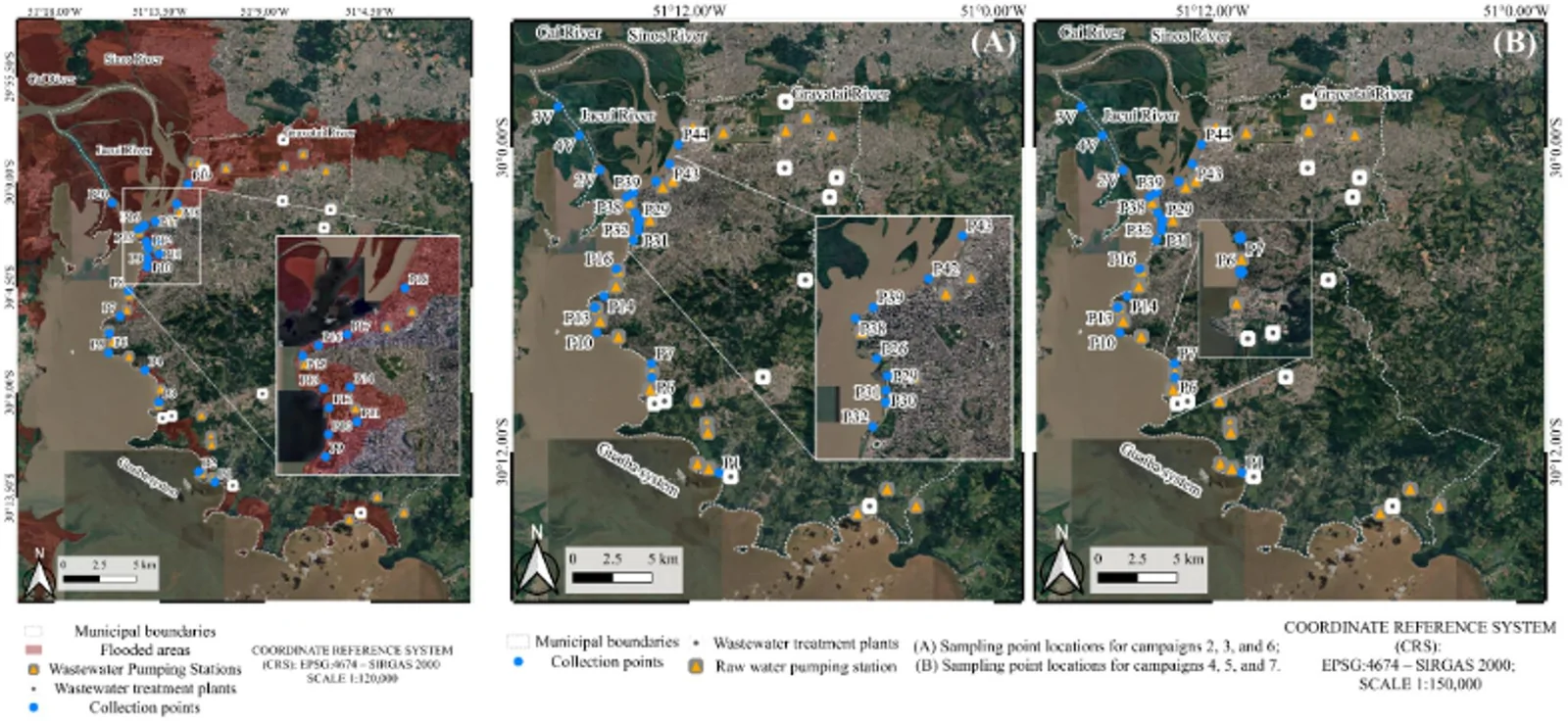
Sampling locations across the Porto Alegre metropolitan region, southern Brazil. The left panel shows the sampling design for Campaign 1, including flooded areas (red polygons), collection points (blue dots), WWPS (orange triangles) and WWTP/WRRF (white squares) locations. The two right panels represent sampling locations for subsequent campaigns: **A** campaigns 2, 3, and 6, and **B** campaigns 4, 5, and 7

During Campaign 1, surface water was collected from 70 distinct sites distributed across Porto Alegre and its metropolitan region, aggregated into 17 composite pools (Fig. 1; Supplementary Table 1). Sampling locations were selected to represent areas strongly affected by flooding, including shoreline zones, urban districts near wastewater infrastructure, and neighborhoods with persistent inundation. All sites were georeferenced (latitude/longitude) and subsequently aggregated into composite samples based on hydrological connectivity and surrounding land-use similarity, as determined in the work of Institute of Hydraulic Research from the Federal University of Rio Grande do Sul (IPH-UFRGS) (Bohnenberger et al., 2026). Composite samples were generated by equal-volume mixing of neighboring raw samples to produce a reduced set of representative samples for virological analysis, described as sample pools in Supplementary Tables 1 and spatially mapped in Fig. 1.

During Campaigns 2–7, sampling focused on surface waters along Guaíba shoreline, from locations where floodwater persisted after the main flood peak, allowing the monitoring of viral dynamics during the recovery phase. Whenever feasible, the same monitoring points were revisited across campaigns to maintain temporal consistency. Each of these campaigns comprised approximately 20–22 samples, with exact sampling locations, geographic coordinates, and spatial distribution maps provided in Supplementary Tables 2 and Fig. 1. This sampling design allowed us to capture both the acute viral contamination pulse associated with the flood event and the subsequent temporal evolution of viral signals during the recovery phase.

### Field Collection, Transport, and Sample Handling Prior to Processing

At each sampling location, 500 mL of flood-impacted surface water was collected into sterile containers using appropriate personal protective equipment. Samples were labeled with unique identifiers and accompanied by field metadata (date/time, location, and GPS coordinates). To minimize cross-contamination, gloves were changed between sampling points and containers were handled aseptically. Samples were transported to the laboratory under refrigeration (2–8 °C) in insulated coolers. Upon arrival, samples were logged and stored at 4 °C until viral concentration. Concentration procedures were initiated after receipt, following the same workflow across campaigns.

### Viral Concentration by Ultracentrifugation

Viral concentration was performed by ultracentrifugation, as previously described, with modifications (Aschidamini Prandi et al., 2022; Girardi et al., 2018; Pina et al., 1998). Briefly, 36 mL of each sample was subjected to ultracentrifugation at 100,000 × g for 1 h at 4 °C. The resulting pellet was resuspended in 4 mL of 0.25 M glycine buffer (pH 9.5) mixed with 2× PBS, followed by clarification by low-speed centrifugation to remove residual debris. The supernatant was then ultracentrifuged again at 100,000 × g for 1 h at 4 °C. The final pellet was resuspended in 0.5 mL of 1× PBS and stored at − 80 °C until RNA extraction.

### Viral RNA Extraction and RT-qPCR Detection

Viral RNA was extracted using the Maxwell^®^ 16 Viral Total Nucleic Acid Purification Kit (Promega, Madison, WI, USA), according to the manufacturer’s instructions. Extracted nucleic acids were stored at − 80 °C until downstream molecular analyses. RT-qPCR detection was performed on a QuantStudio 1 Real-Time PCR System using AgPath-ID™ One-Step RT-PCR Kit (Thermo Fisher Scientific), in a final reaction volume of 15 µL, including 5 µL of RNA template or Nuclease-Free H2O as non-template control (NTC). Cycling conditions followed the AgPath user guide: one cycle of 50 °C for 15 min (reverse transcription), 96 °C for 10 min (initial denaturation), followed by 45 cycles of 96 °C for 10 s (denaturation) and assay-specific temperatures for annealing in each target. Specific primers and probes were used to detect genomic targets of the screened pathogens: the HAV 5′-UTR region (Jothikumar et al., 2005), HEV ORF3 region (Jothikumar et al., 2006), norovirus genogroups I and II (NoV GI and GII) ORF1-2 junction regions (Kageyama et al., 2003), and the SARS-CoV-2 N1 genomic region (CDC, 2020). Primer and probe sequences are provided in Supplementary Table 3. All samples were analyzed in technical duplicates. A target was considered detected when amplification was observed in both replicates, and the reported Ct corresponds to the mean of the positive replicates; samples with discordant replicates were re-tested. Each RT-qPCR run included a non-template control and target-specific positive controls to monitor assay performance and potential cross-contamination. Composite pools were concentrated, extracted and amplified under the same conditions as the individual samples. A sample was considered positive when amplification was observed with a cycle threshold (Ct) < 40, consistent with previously published wastewater RT-qPCR studies (Randazzo et al., 2020).

### SARS-CoV-2 RNA Quantification

SARS-CoV-2 RNA concentrations were quantified using a standard curve–based RT-qPCR approach. Standard curves were generated from serial 10-fold dilutions (five points) of a SARS-CoV-2 positive control previously quantified by digital droplet PCR (ddPCR), a quantification approach previously applied to waterborne viruses (Monteiro & Santos, 2017). This control was established and validated for environmental and wastewater matrices in our laboratory during routine SARS-CoV-2 wastewater surveillance. Ct values obtained from environmental samples were interpolated against the standard curve, and SARS-CoV-2 RNA loads were calculated accounting for the processed sample volume (36 mL), concentration steps and elution volumes (Aschidamini Prandi et al., 2022); the calibration curve linear equation, amplification efficiency, and R^2^ values are detailed in Supplementary Fig. 1 (S1). Therefore, results were expressed as genome copies per liter (GC L⁻¹).

### Data Organization and Reporting

For each sampling campaign, viral detection results were summarized by target virus (SARS-CoV-2, norovirus GI, norovirus GII, hepatitis A virus [HAV], and hepatitis E virus [HEV]) as detection frequency across samples, expressed as the proportion of positive samples per campaign. For SARS-CoV-2, both detection frequency and RNA concentration (GC L⁻¹) were used to describe temporal patterns across the seven sampling campaigns, from the acute flood phase to the post-flood recovery period. Summary visualizations, including frequency-based plots and temporal concentration trends for SARS-CoV-2, were used to facilitate comparison of viral occurrence across time and sampling phases.

Where publicly available data allowed, viral detection patterns were compared with epidemiological indicators from local and national health surveillance systems. For HAV, confirmed case data retrieved from the national reporting system, Notifiable Diseases Information System (SINAN), were used to contextualize the observed increase in environmental detections during the post-flood period (Ministério da Saúde, 2026). For SARS-CoV-2, RNA concentrations were compared with notified case counts from the state surveillance system (CEVS, 2025). No formal comparisons were performed for norovirus or HEV, given the absence of routine clinical surveillance data for these pathogens in Brazil.

### Statistical Analysis

Detection frequencies were compared between individual campaigns with Fisher’s exact test, and between the three sampling phases (flood, Campaigns 1–3; recovery, Campaigns 4–6; one year, Campaign 7) by Monte-Carlo permutation of the corresponding 3 × 2 contingency tables. Temporal trend was assessed with the Cochran–Armitage trend test, in which trend scores corresponded to the number of days elapsed since the flood peak (0, 13, 27, 51, 112, 205 and 355 days); trend results were confirmed by Monte-Carlo permutation (50,000 iterations). Cycle-threshold (Ct) values, used as a relative proxy of viral load, were compared with the Mann–Whitney U and Kruskal–Wallis tests. The association between SARS-CoV-2 environmental detection frequency and notified COVID-19 cases was assessed with Spearman’s rank correlation; given the small number of campaigns (*n* = 7), this comparison is exploratory. Multiple comparisons were controlled with the Benjamini–Hochberg false-discovery-rate procedure. All tests were two-sided with α = 0.05 and were performed in Python 3 (SciPy 1.18).

## Results

### Overview of Viral Detection Across Seven Sampling Campaigns

**Fig. 2:**
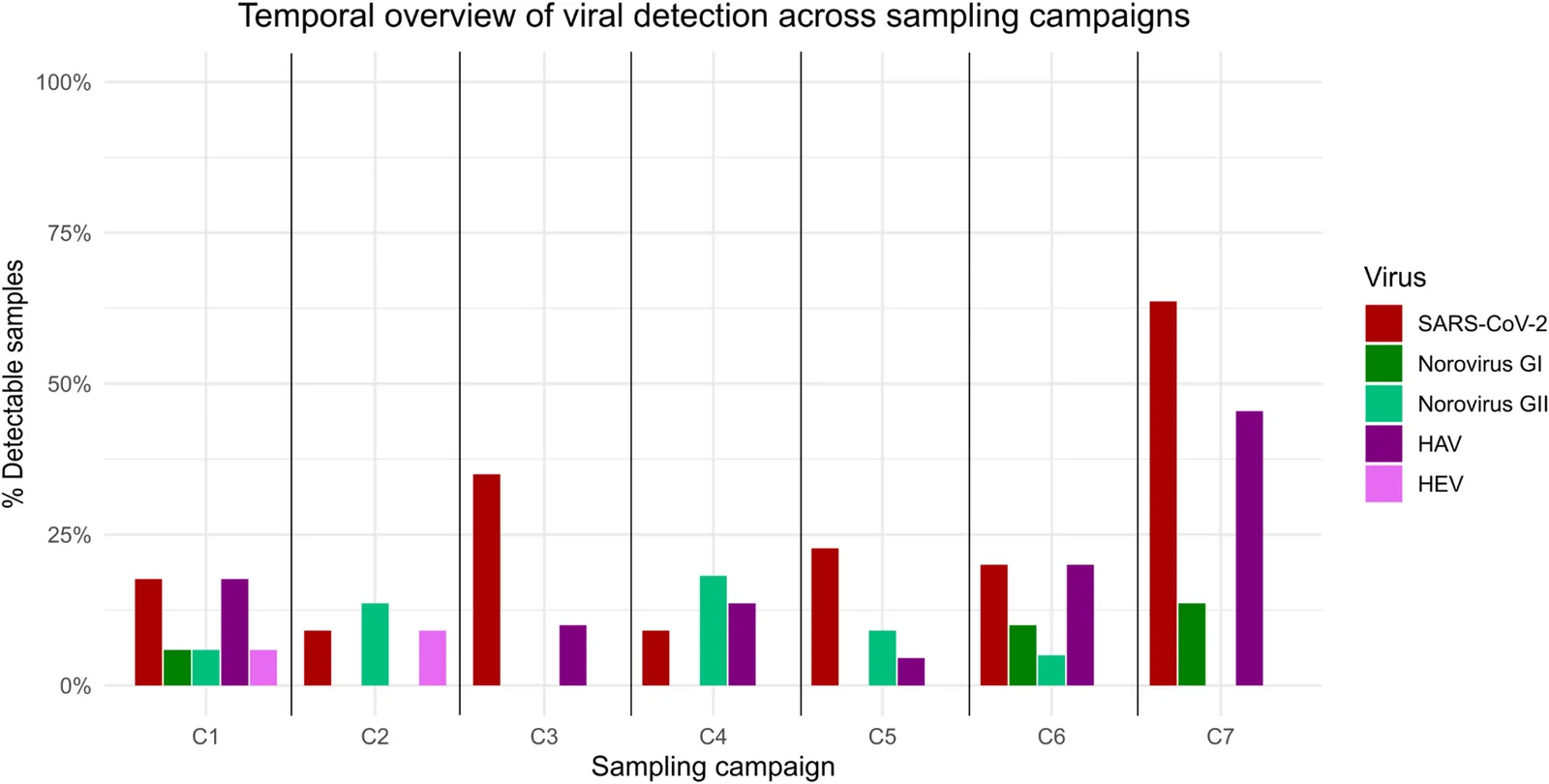
Proportion of RT-qPCR–positive samples for each viral target by sampling campaign. Grouped bar plot showing the percentage of positive samples for SARS-CoV-2, norovirus GI, norovirus GII, HAV, and HEV across seven sampling campaigns performed over a one-year monitoring period, respectively. For each campaign, detection frequency was calculated relative to the total number of samples analyzed. The plot enables direct comparison of temporal variation in viral occurrence and diversity across campaigns

Across the seven sampling campaigns conducted over approximately one year, all five targeted viral pathogens (SARS-CoV-2, norovirus GI, norovirus GII, hepatitis A virus [HAV], and hepatitis E virus [HEV]) were detected in at least one sampling campaign. Viral detection patterns varied markedly over time, reflecting a transition from an acute flood-associated contamination scenario to a background pattern of persistent detection of viral genomic material in flood-impacted surface waters (Fig. 2).

**Fig. 3:**
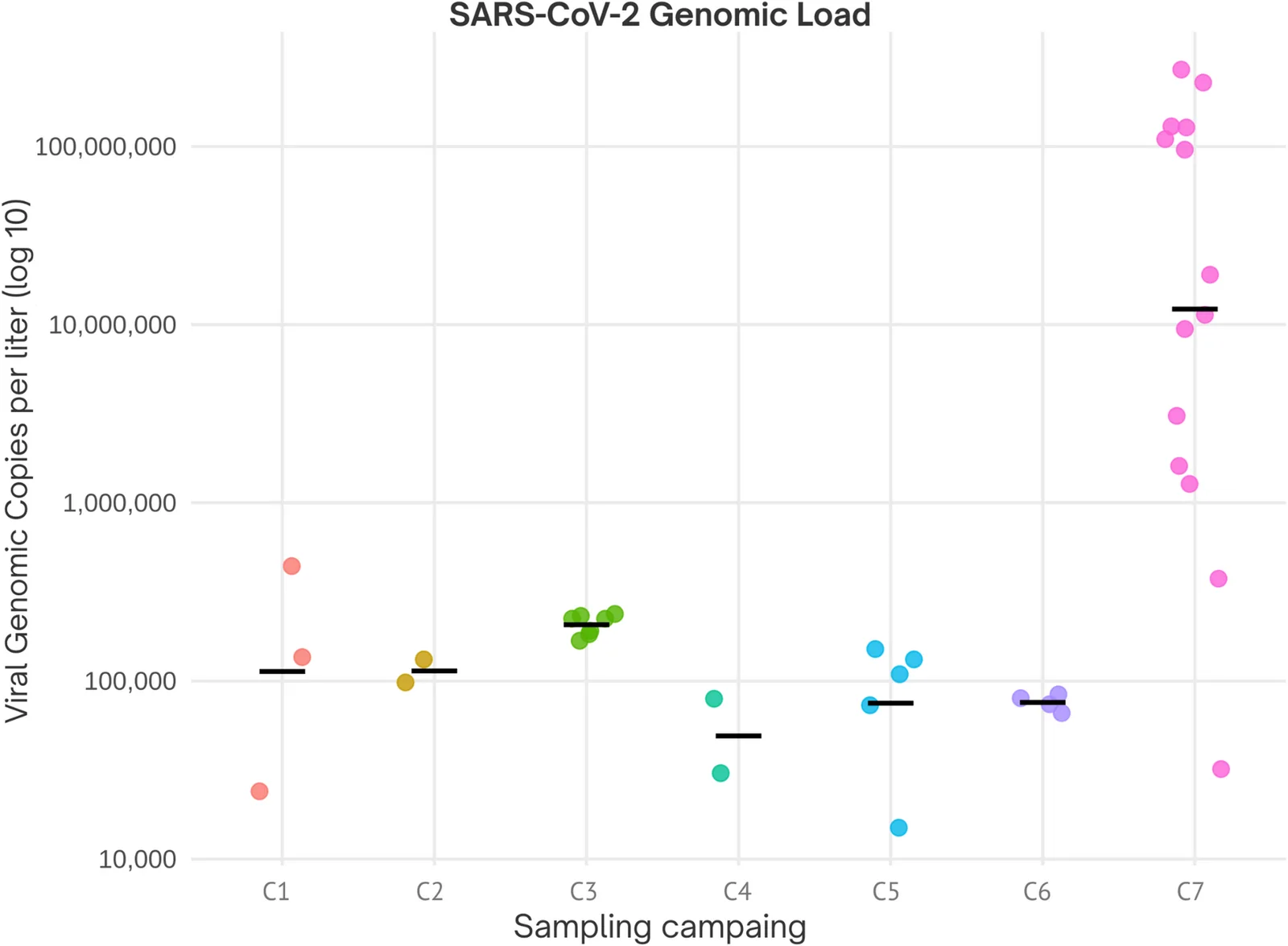
SARS-CoV-2 RNA concentration across consecutive sampling campaigns. Viral quantification is expressed in genomic copies per liter (GC/L). Each data point represents an individual positive sample within its respective campaign (C1 to C7). Bars indicate the mean viral concentration for each campaign

SARS-CoV-2 was the most frequently detected virus, being identified in all seven campaigns, although in a minority of sampling locations in every campaign except the last (C7, 14/22, 63.6%), and showing distinct patterns of genomic load (Fig. 3). Norovirus genogroups I and II exhibited greater temporal variability: GII was detected intermittently from the flood peak up to Campaign 6, whereas GI was detected at the flood peak, was absent from Campaigns 2 to 5, and reappeared in Campaigns 6 and 7. HEV was detected exclusively at the flood peak. By the final campaign, conducted approximately one year after the flood, only SARS-CoV-2, HAV, and norovirus GI were detected.

### Viral Detection During the Acute Flood Phase

**Fig. 4:**
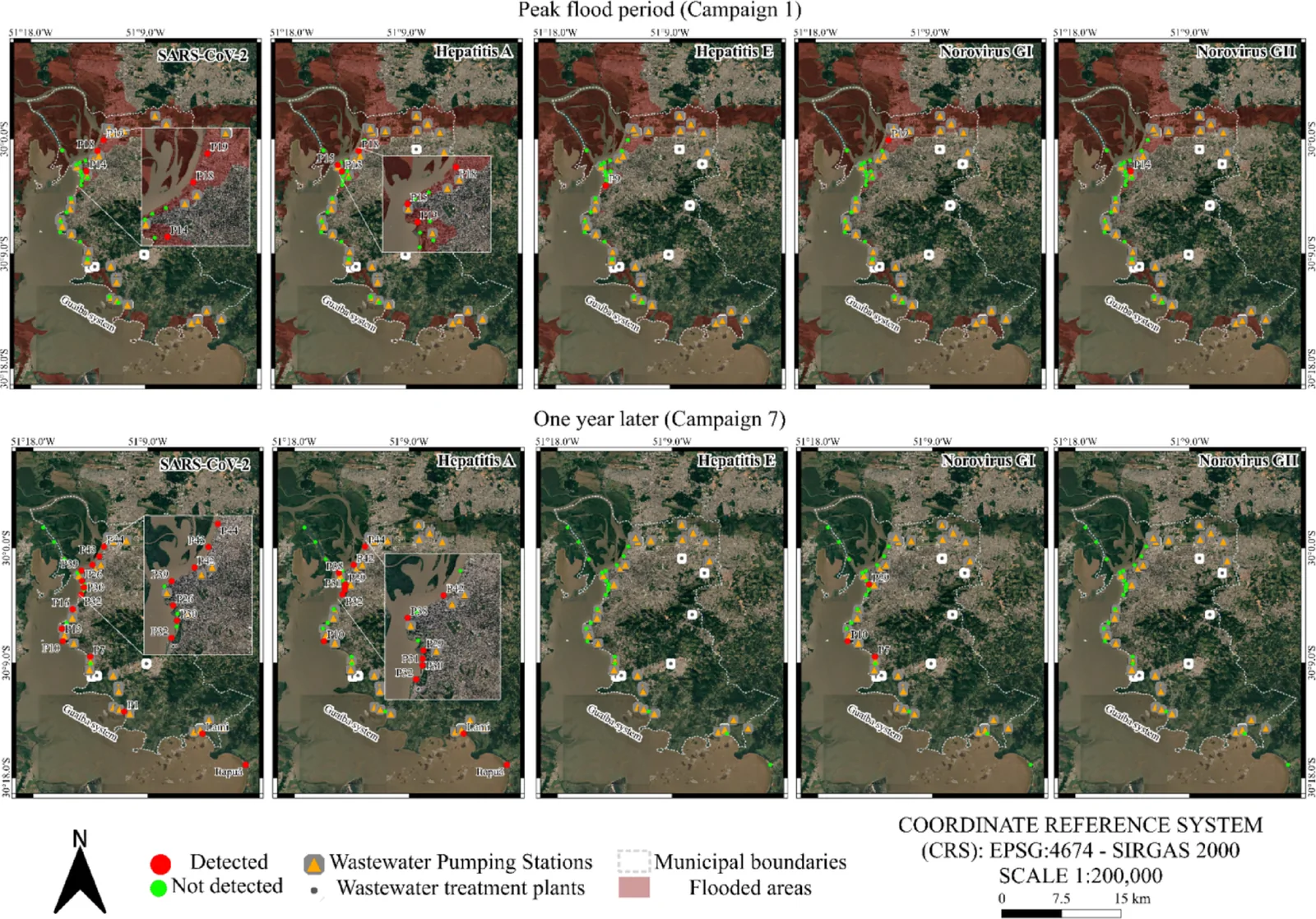
Comparison of viral detection patterns during the peak flood period (Campaign 1, top panels) and one year after the event (Campaign 7, bottom panels) across the Porto Alegre metropolitan region, southern Brazil. Each column represents a different viral target (SARS-CoV-2, hepatitis A virus [HAV], hepatitis E virus [HEV], norovirus GI, and norovirus GII, respectively). Sampling points are classified as detected (red) or not detected (green). WWPS locations are shown as orange triangles and WWTP/WRRFs are shown by gray dots inside white squares. Flooded areas during the peak event are shown as red polygons, and municipal boundaries are indicated by dashed lines. Insets highlight the central urban area at higher spatial resolution. The figure illustrates the shift from widespread multi-virus detection during the flood to a more persistent detection pattern one year later, characterized by the continued presence of SARS-CoV-2, the increased detection of HAV, and the reduced detection of norovirus GI, with the absence of HEV and norovirus GII

During the peak flood period (Campaign 1), all targeted viral pathogens were detected in at least one sampling location (Fig. 4). SARS-CoV-2 and HAV were the most prevalent viruses, with 17.6% (3/17) of samples positive for each, being detected in collections from the Historic Centre, where floodwaters directly entered urban areas, and from sites along the northern margin of Guaíba System, which could indicate widespread contamination likely associated with wastewater inputs (Supplementary Table 4; Fig. 4).

Norovirus GI was detected in a sample (1/17–5.88%) collected in close proximity to the São João Water Resource Recovery Facility (WRRF), located in the Navegantes neighborhood, one of the areas most severely impacted by the flooding event. Norovirus GII was detected in a central urban area (1/17–5.88%), while HEV was identified in a sample from the southern shoreline region (1/17–5.88%) (Fig. 4).

Spatial characterization revealed that 58.8% (10/17) of the analyzed composite samples contained at least one individual collection site in close proximity to WRRFs or wastewater pumping stations (WWPSs) that were rendered inoperative during the flood (Fig. 1; Supplementary Table 1). Eight of the nine viral signals detected in this campaign exhibited high cycle threshold values (Ct > 30), indicating low viral RNA abundance in the samples analyzed (Supplementary Table 4). In contrast, the Norovirus GI–positive composite sample (19P), collected between the non-operational São João WRRF and the São João/Moinhos de Vento WWPS, exhibited a substantially lower Ct value (Ct < 23).

### Temporal Evolution of Viral Detection During Post-flood Recovery

Following the acute flood phase, the diversity and frequency of viral detections fluctuated progressively across subsequent campaigns. Campaigns 2 and 3 (May 21 st and June 4th, 2024) corresponded to the period when floodwaters were still present in the urban area, whereas subsequent campaigns, Campaign 4 to Campaign 6 (June 28th to November 29th, 2024), corresponded to the post-flood recovery phase. HEV was detected in a single flood-peak sample (1/17 in C1) and in no subsequent campaign. Norovirus GI followed a discontinuous pattern, with one detection at the flood peak (1/17 in C1), no detection between C2 and C5 (0/20, 0/20, 0/22 and 0/22, respectively), and a reappearance in C6 (2/20). Norovirus GII was detected intermittently, with frequencies of 3/20 (C2), 0/20 (C3), 4/22 (C4), 2/22 (C5) and 1/20 (C6), and showed no statistically significant temporal pattern (*p* = 0.13). SARS-CoV-2 and HAV continued to be detected across multiple campaigns (Supplementary Table 4), with frequencies of 2/20 (C2), 5/20 (C3), 2/22 (C4), 5/22 (C5) and 4/20 (C6) for SARS-CoV-2, and 0/20 (C2), 2/20 (C3), 3/22 (C4), 1/22 (C5) and 4/20 (C6) for HAV.

Across Campaigns 2 to 6, detection frequencies ranged from 9.1% to 25.0% for SARS-CoV-2 and from 0% to 20.0% for HAV, values comparable to those recorded at the flood peak (17.6% for both targets).

### Viral Detection One Year After the Flood

One year after the flood event (Campaign 7, April 28th, 2025), three of the five targeted viral pathogens were detected (SARS-CoV-2, HAV and Norovirus GI), while norovirus GII and HEV yielded negative results in all collected samples. SARS-CoV-2 was the most frequently detected target, present in the majority of the sampling points (14/22) (Fig. 4). SARS-CoV-2 RNA detection increased significantly over the monitoring period (Cochran–Armitage *p* < 0.001), an increase concentrated in the final campaign: detection in C7 (14/22, 63.6%) was significantly higher than in Campaigns 1–6 combined (21/121, 17.4%; Fisher’s exact *p* < 0.001), whereas detection across Campaigns 1–6 showed no significant trend (*p* = 0.56). This was accompanied by significantly higher viral load in C7, reflected in lower Ct values (median Ct 29.7 vs. 36.8 in earlier campaigns; Mann–Whitney *p* < 0.001; Kruskal–Wallis across campaigns *p* = 0.001). Despite the elevated viral RNA concentrations obtained by quantification, no significant correlation was found between SARS-CoV-2 environmental detection frequency and notified COVID-19 cases across the seven campaigns (Spearman ρ = 0.23, *p* = 0.61), reinforcing that environmental signal and clinical notification were decoupled during the study period.

HAV detection also increased significantly over time (Cochran–Armitage *p* < 0.0005; C7 vs. earlier campaigns Fisher’s exact *p* = 0.001), although the isolated comparison of the acute flood period (Campaign 1) vs. the scenario one year later (Campaign 7) did not reach significance (3/17 vs. 9/22; Fisher’s exact *p* = 0.17) (Fig. 4). All contrasts reported above remained significant after Benjamini–Hochberg correction, with the exception of the isolated Campaign 1 versus Campaign 7 comparison for HAV (adjusted *p* = 0.24). HAV Ct values did not differ significantly across campaigns (*p* = 0.53), indicating that the change was in detection frequency rather than viral load. This increase in HAV detection frequency aligns with epidemiological trends reported by the Municipal Health Department, which formally declared an outbreak of hepatitis A in the municipality during this post-flood period. While absolute clinical numbers are heavily limited by underreporting, the official recognition of an active outbreak corroborates the widespread environmental dissemination observed in our study.

In contrast, norovirus GI exhibited a more restricted distribution, occurring at only 3/22 of the sites, indicating a more localized environmental presence.

When campaigns were grouped into the three phases described above, detection frequency differed significantly between phases for SARS-CoV-2 (10/57 during the flood phase, 11/64 during recovery and 14/22 one year later; permutation *p* < 0.001) and for HAV (5/57, 8/64 and 9/22, respectively; *p* = 0.001), in both cases driven by the final campaign: the flood and recovery phases did not differ from each other (SARS-CoV-2, 17.5% versus 17.2%; HAV, 8.8% versus 12.5%). No significant difference between phases was detected for norovirus GI (*p* = 0.06), norovirus GII (*p* = 0.22) or HEV (*p* = 0.55). The acute flood phase was therefore distinguished by the simultaneous detection of all five targets rather than by a higher per-target detection frequency. Overall, the results reveal a transition from an acute flood-associated contamination event, characterized by the simultaneous detection of all five targets, to a persistent detection of viral genomic material dominated by SARS-CoV-2, HAV, and norovirus GI. This pattern indicates that, even after hydrological normalization, flood-impacted urban waters continue to yield viral genomic signals.

## Discussion

### Acute Viral Contamination and Sanitary Infrastructure Collapse During the Flood

The acute flood phase revealed the simultaneous detection of all five targeted viral pathogens, indicating a multi-source contamination pulse that could have been driven by the widespread failure of urban drainage and wastewater infrastructure. What makes this case particularly striking is the scale at which infrastructure collapse unfolded: within days, five of Porto Alegre’s six water treatment plants were offline, stormwater pumps were deliberately shut down to prevent irreparable damage, and raw wastewater entered the Jacuí River at an estimated rate of 50,000 m³ per day, exposing hundreds of thousands of residents directly (Albornoz et al., 2026). In this context, the release and redistribution of untreated wastewater into surface waters was not incidental, it was systemic and inevitable. Similar contamination pulses have been documented in other flood-affected cities where combined sewer systems are overwhelmed, but the simultaneous failure of multiple infrastructure nodes here produced conditions of unusual severity (Han & He, 2021; Ten Veldhuis et al., 2010).

Most viral detections during the flood peak exhibited high Ct values (> 30), consistent with the massive hydrological dilution that characterized this event, as the Guaíba system reached an unprecedented 5.35 m level and inundated roughly 4,300 km² of the surrounding region (Albornoz et al., 2026). High dilution is expected in such scenarios and has been reported in other flood-related environmental surveillance studies (Andrade et al., 2018; Kraay et al., 2020). When interpreting these early signals, it is important to note that samples from Campaign 1 were analyzed as composite pools (representing grouped sampling locations), whereas Campaigns 2 through 7 evaluated individual raw surface water samples; this spatial aggregation during the acute peak may have further influenced detection thresholds. Nevertheless, the localized detection of norovirus GI with low Ct values (< 23) near a water resource recovery facility (WRRF) suggests that point-source wastewater overflows can generate high viral loads even under extensive dilution, reinforcing the importance of spatial context when interpreting environmental viral signals (Bullen et al., 2025; Farkas et al., 2018). These findings support the notion that floodwaters act not only as diluents but also as efficient dispersal media for human viruses under conditions of infrastructural stress. Disaster conditions also compound the surveillance gap on the clinical side: during and immediately after the flood, health services were overwhelmed, over 1,300 leptospirosis cases were registered between May and July 2024 alone (Albornoz et al., 2026), and etiological testing for gastroenteric illness fell predictably low on clinical priorities. The Brazilian public health system does not routinely confirm norovirus or HEV in clinical samples, making direct epidemiological comparisons for these pathogens unfeasible.

### Transition from Acute Contamination to Persistent Environmental Detection

As floodwaters receded, the virological picture changed gradually but clearly. The initial multi-pathogen pulse gave way to a narrower set of persisting signals, tracing a transition from the acute contamination observed during the flood peak to the persistent detection of viral genomic material throughout the monitoring period. HEV was confined to the flood peak and norovirus GII was detected intermittently up to Campaign 6, without a statistically significant temporal pattern, whereas SARS-CoV-2 and HAV were detected in all or most campaigns and norovirus GI reappeared in the final two campaigns. This temporal stratification suggests differential environmental persistence and ongoing input mechanisms among viral groups, as previously documented in wastewater-based surveillance studies (Farkas et al., 2018; Smith et al., 2024).

SARS-CoV-2 remained detectable across all campaigns, consistent with its widespread circulation in the human population and continuous shedding into wastewater systems, even outside epidemic peaks (Li et al., 2022). The persistence of norovirus GI, in contrast to the disappearance of GII, may reflect differences in genogroup community circulation prevalence, environmental stability, or stochastic introduction patterns, as suggested by prior environmental and wastewater studies (Fioretti et al., 2011). These observations underscore the importance of multi-virus monitoring to disentangle flood-specific contamination from endemic background signals.

Beyond acute dissemination, the persistent detection of viral targets across the one-year monitoring period must be interpreted critically alongside alternative environmental and hydrological mechanisms. Rather than solely reflecting sustained active community transmission, persistent molecular signals can be driven by the prolonged environmental persistence of viral nucleic acids, which are known to remain detectable via RT-qPCR long after loss of infectivity, particularly when protected within organic aggregates or sediments (Rames et al., 2016; Staggemeier et al., 2015). Subsequent hydrological fluctuations and sediment resuspension during high-flow events can remobilize this bound viral RNA back into the water column, mimicking new contamination events (Farkas et al., 2018). Furthermore, seasonal variations in water temperature, solar UV irradiation, and precipitation dynamics substantially influence viral decay rates and runoff inputs throughout the year. Continuous, unmonitored raw sewage discharges from non-centralized urban areas into the Guaíba basin also maintain a baseline viral input. Indeed, pre-disaster surveillance in southern Brazilian surface waters has documented a high baseline viral burden driven by chronic sanitation deficits. Within the Guaíba hydrographic region, historical monitoring in the Sinos River basin, one of the system’s primary tributaries, revealed the widespread circulation of human adenoviruses and enteric viruses across urban abstraction points (Dalla Vecchia et al., 2015). Because longitudinal virological monitoring in the Guaíba basin remained scarce in the years preceding the 2024 disaster, broader investigations in neighboring southern Brazilian catchments provide essential regional context; studies in Santa Catarina, for instance, documented the pervasive occurrence of noroviruses, rotaviruses, and hepatitis A virus in impacted rivers and surface waters linked to domestic wastewater discharges (Cadamuro et al., 2021; Mortari et al., 2023).

Furthermore, our results corroborate a persistent background detection of viral genomic material of SARS-CoV-2 in the Porto Alegre metropolitan region. However, a direct correlation between the high genomic loads detected one year after the flood event and an increase in COVID-19 cases could not be established. Officially reported clinical cases remained low during our sampling periods (Secretaria de Saúde - RS, 2024a). This discrepancy could be caused by an underreporting of cases, as the availability of rapid self-testing and the tendency to manage mild symptoms at home can mask the true community prevalence.

### Increasing HAV Detection and Its Public Health Relevance

Epidemiological reports for Hepatitis A show that Porto Alegre recorded a detection rate of 8.6 cases per 100,000 inhabitants in 2024, the fourth highest HAV incidence among Brazilian state capitals (Ministério da Saúde, 2025). Our findings show that the HAV genome was detected in six of the seven campaigns carried out between May 2024 and April 2025, consistent with a continued environmental input of this pathogen in the metropolitan region.

Among the viruses monitored, HAV showed the clearest temporal change: detection frequency increased significantly across the monitoring period (Cochran–Armitage *p* < 0.0005), with detection in the final campaign significantly higher than in all earlier campaigns combined (9/22 versus 13/121; Fisher’s exact *p* = 0.001). Two features of this increase deserve emphasis. First, it was an increase in detection frequency and not in viral load, since HAV Ct values did not differ significantly across campaigns (*p* = 0.53). Second, the isolated comparison between the flood peak and one year later did not reach significance (3/17 versus 9/22; *p* = 0.17) and, because Campaign 1 samples were analyzed as composite pools, the pattern should be read as an increase in environmental detection rather than as a demonstrated expansion in the number of contaminated sites. Far from fading after the flood, therefore, the HAV signal became progressively more frequent, a pattern that is difficult to attribute solely to residual contamination from the flood itself and that is compatible with continued fecal-oral viral input into the urban water system, although environmental persistence and remobilization of viral nucleic acids cannot be excluded. This environmental signal was accompanied by contemporaneous increases in clinically reported hepatitis A cases, with 193 confirmed cases by July 2025 (Ministério da Saúde, 2026), and by the formal declaration of an outbreak by the Municipal Health Department, reinforcing the value of environmental surveillance as a complementary tool for public health monitoring (Secretaria de Saúde - RS 2024b).

Previous studies have shown that wastewater surveillance can detect HAV circulation even during periods of limited or delayed clinical reporting, often providing early or independent evidence of community transmission (Hellmér et al., 2014; La Rosa et al., 2014). In this context, the observed increase in HAV detection is consistent with continued viral input into the urban water system, sustained by chronic gaps in sanitation infrastructure that long predate and will outlast the 2024 event. In this sense, extreme flood events operate as involuntary stress tests of urban sanitation, exposing the baseline fragility that routine conditions are able to conceal.

### Implications for Surface Water Surveillance and One Health Approaches

Each phase acute, transitional, and chronic carries distinct surveillance priorities, and a monitoring design that ends with the emergency will miss the longer story. From a One Health perspective, these findings underscore the interconnectedness of climate extremes, sanitation infrastructure, and public health risk. The Porto Alegre event illustrates this with unusual clarity: the same infrastructure failures described above simultaneously created the conditions for widespread viral dispersal across the urban water matrix (Albornoz et al., 2026; Collischonn et al., 2025). Wastewater and environmental viral surveillance, if embedded in disaster response protocols, could serve as an early-warning layer during the acute phase and as a recovery-tracking tool in the months that follow precisely the temporal coverage this study sought to provide. In cities like Porto Alegre, where extreme rainfall events are already intensifying (Marengo et al., 2021; Rathore et al., 2025) and sanitation infrastructure remains incomplete in many neighborhoods, the argument for institutionalizing such surveillance is not speculative, it is grounded in what this flood showed us.

## Conclusion and Perspectives

This study presents a one-year longitudinal virological surveillance of flood-impacted surface waters following the May 2024 flood in Porto Alegre, Brazil. By tracking five viral targets across seven campaigns, we documented a clear two-phase contamination dynamic: an acute multi-pathogen pulse during the flood peak, driven by the simultaneous collapse of drainage, wastewater, and water supply infrastructure, followed by a phase of persistent detection dominated by SARS-CoV-2, hepatitis A virus, and norovirus GI. Perhaps the most consequential finding is the statistically significant increase in HAV detection frequency over the monitoring period, corroborated by epidemiological records showing a marked rise in reported hepatitis A cases, which indicates that the environmental input of HAV into the urban water system persisted well beyond the acute emergency. This challenges any assumption that public health risk ends when floodwaters recede: in cities with pre-existing sanitation gaps, the effects of an extreme flood on human health are not bounded by the event itself. Recovery, in this sense, is incomplete until the underlying structural deficits are addressed.

Some limitations should be noted. First, the whole-process recovery controls and PCR-inhibition assessments were not incorporated into the analytical protocol; consequently, false negatives cannot be excluded, and all values reported here represent detection frequencies rather than true biological prevalences. Second, although the five targeted viruses represent clinically relevant pathogens, they capture only a fraction of the viral diversity potentially mobilized during a flood event of this magnitude. Third, samples from the first campaign were analyzed as composite pools, whereas subsequent campaigns evaluated individual raw surface water samples; this spatial aggregation reduces site-level resolution during the acute peak, meaning hotspot interpretations for the flood peak must be treated with caution and are not directly comparable to later campaigns. Fourth, the lack of genomic quantification for targets beyond SARS-CoV-2 limits temporal comparisons of relative viral abundance. Fifth, RT-qPCR detects viral genomic material and cannot distinguish infectious particles from degraded or remobilized environmental RNA; our findings therefore document viral genome presence rather than active infectivity or direct transmission risk. Finally, the absence of routine epidemiological surveillance for norovirus and HEV in Brazil also makes it impossible to triangulate environmental signals against clinical trends for those pathogens.

Looking forward, virome-wide sequencing of the archived samples collected during this study would provide an unbiased view of the full viral community mobilized by the 2024 flood, an approach we intend to pursue, and one that could both validate and substantially extend the targeted findings reported here. Broader epidemiological surveillance coverage, particularly for enteric viruses, would help close the loop between environmental signals and clinical outcomes. More immediately, the surveillance framework developed here should inform emergency preparedness planning in Porto Alegre and comparable cities in the region. The question is no longer whether events like 2024 will happen again, as the evidence suggests they will. The question is whether the public health system will be ready to detect and respond to their pathogen consequences from the first hours through the final months.

## Supplementary Information

Below is the link to the electronic supplementary material.

Supplementary Material 1

## Data Availability

No datasets were generated or analysed during the current study.
