# Supplementary material for: Longitudinal Surface Water Viral Surveillance Following the 2024 Extreme Flood: One-Year Monitoring in Porto Alegre, Brazil
Source: Food Environ Virol. 2026 Oct 26;18(4):39. doi: 10.1007/s12560-026-09713-2 (PMC13710650; doi:10.1007/s12560-026-09713-2)
Supplement: Supplementary file 1 [file 12560_2026_9713_MOESM1_ESM.docx]

**SUPPLEMENTARY INFORMATION FOR**

**Longitudinal Surface Water Viral Surveillance Following the 2024 Extreme Flood: One-Year Monitoring in Porto Alegre, Brazil**

Thales De Lima Bermann^a,b,#^, Fernanda Mückler-Pereira^a,#,*^, Bruno Aschidamini Prandi^a^, Nicóle Verardo De Mattos^a^, Vítor Felippi Tessele^a^, Luis Gustavo Dos Santos Da Silva^a^, Nicole Vieira Stone^a^, Raissa Gasparetto^a^, Bruna Simone Paredes-Galarza^a^, Martha Trindade Oliveira^a^, Louidi Lauer Albornoz^c^, Lucas Bohnenberger^d^, Salatiel W. da Silva^d^, Ana Cláudia Franco^a^.

^a^ Virology Laboratory, Microbiology, Immunology and Parasitology Department, Institute of Basic Health Sciences, Federal University of Rio Grande do Sul, Porto Alegre, Brazil.

^b^ Genetics Department, Institute of Biosciences, Post-Graduate Program in Genetics and Molecular Biology (PPGBM), Federal University of Rio Grande do Sul, Porto Alegre, Brazil.
^c^ Center of Environmental Sanitation Studies (NESA) and Post-Graduate Program in Water Resources and Environmental Sanitation (PPG-IPH), Porto Alegre, Brazil.

^#^ Thales L. Bermann and Fernanda Mückler-Pereira, equal first-author contribution

*Corresponding author: Fernanda Mückler-Pereira

E-mail: fmucklerp@gmail.com, ORCID: <https://orcid.org/0009-0006-0405-8789>.

Address: Virology Laboratory, Microbiology, Immunology and Parasitology Department, Institute of Basic Health Sciences, Federal University of Rio Grande do Sul (UFRGS). Ramiro Barcelos St. 2600, Porto Alegre, Brazil.

Methods

Table S1 presents the geographical coordinates for the 70 collection sites of the first campaign, during acute flooding, and their corresponding composite sample pooling (17 pools).

Supplementary Table 1. Geographical coordinates of Campaign 1 collection sites and composite sample pooling identifications.

| **Raw Sample** | **Coordinates** | **Composite pool** | **Sampling points pooled** |
| --- | --- | --- | --- |
| 1M | 30°01'37"S 51°13'21"W | 1RP | **P1** and P2 |
| 2M | 30°01'40"S 51°13'28"W | 3P | P4, P5 and **P6** |
| 3M | 30°01'43"S 51°13'32"W | 4P | **P7** and P8 |
| 4M | 30°01'44"S 51°13'36"W | 5P | P9, **P10** and P11 |
| 5M | 30°01'52"S 51°13'57"W | 6P | P12, P45 and **P13** |
| 6M | 30°01'53"S 51°14'04"W | 8P | **P16** and P34 |
| 8M | 30°01'52"S 51°14'09"W | 9P | P17, 18, **32** and 33 |
| 9M | 30°01'56"S 51°14'13"W | 10P | **P30** and **31** |
| 10M | 30°01'59"S 51°14'20"W | 11P | P19, 1L, 2L, 3L and 4L |
| 11M | 30°01'57"S 51°14'19"W | 13P | P28, P27, P35, P25, **P26** |
| 5L | 30°02'37"S 51°13'13"W | 14P | P20, 5L, P21 and P22 |
| 4L | 30°02'58"S 51°13'26"W | 15P | P36, P24, P37, P23 and P38 |
| 3L | 30°02'58"S 51°13'30"W | 16P | 10M, 11M, 9M, 8M, 6M, 5M, 7V, **P39** and P40 |
| 2L | 30°03'02"S 51°13'32"W | 17P | P41, 10N, 4M, 3M, 2M, 11N, 1M |
| 1L | 30°03'11"S 51°13'34"W | 18P | **P42**, 6V, 2J, 1J |
| P45 | 30°06'37"S 51°15'27"W | 19P | **P43**, 1N, 2N, 3N, 9N and **P44** |
| P44 | 29°59'52"S 51°12'27"W | 20RP | **3V**, **4V**, **2V** and 1V |
| P43 | 30°00'38"S 51°12'48"W |  |  |
| P42 | 30°01'19"S 51°13'20"W |  |  |
| P41 | 30°01'32"S 51°13'45"W |  |  |
| P40 | 30°01'38"S 51°13'56"W |  |  |
| P39 | 30°01'46"S 51°14'13"W |  |  |
| P38 | 30°01'56"S 51°14'30"W |  |  |
| P37 | 30°02'06"S 51°14'34"W |  |  |
| P36 | 30°02'30"S 51°14'31"W |  |  |
| P35 | 30°02'44"S 51°14'00"W |  |  |
| P34 | 30°04'27"S 51°14'35"W |  |  |
| P33 | 30°03'48"S 51°14'23"W |  |  |
| P32 | 30°03'39"S 51°14'13"W |  |  |
| P31 | 30°03'16"S 51°14'01"W |  |  |
| P30 | 30°03'04"S 51°14'01"W |  |  |
| P28 | 30°02'48"S 51°14'04"W |  |  |
| P27 | 30°02'43"S 51°14'09"W |  |  |
| P26 | 30°02'34"S 51°14'09"W |  |  |
| P25 | 30°02'33"S 51°14'07"W |  |  |
| P24 | 30°02'12"S 51°14'27"W |  |  |
| P23 | 30°02'08"S 51°14'24"W |  |  |
| P22 | 30°02'17"S 51°13'37"W |  |  |
| P21 | 30°02'23"S 51°13'20"W |  |  |
| P20 | 30°02'23"S 51°13'20"W |  |  |
| P19 | 30°03'17"S 51°13'29"W |  |  |
| P18 | 30°03'39"S 51°13'40"W |  |  |
| P17 | 30°03'53"S 51°13'55"W |  |  |
| P16 | 30°04'47"S 51°14'54"W |  |  |
| P13 | 30°06'19"S 51°15'45"W |  |  |
| P12 | 30°06'46"S 51°15'26"W |  |  |
| P11 | 30°06'53"S 51°15'27"W |  |  |
| P10 | 30°07'18"S 51°15'40"W |  |  |
| P9 | 30°07'24"S 51°15'20"W |  |  |
| P8 | 30°08'06"S 51°13'42"W |  |  |
| P7 | 30°08'31"S 51°13'30"W |  |  |
| P6 | 30°09'03"S 51°13'29"W |  |  |
| P5 | 30°09'11"S 51°13'22"W |  |  |
| P4 | 30°09'24"S 51°13'21"W |  |  |
| P2 | 30°12'28"S 51°11'31"W |  |  |
| P1 | 30°12'49"S 51°10'51"W |  |  |
| 1V | 30°00'54"S 51°15'35"W |  |  |
| 2V | 30°00'53"S 51°15'32"W |  |  |
| 3V | 29°58'23"S 51°17'12"W |  |  |
| 4V | 29°59'31"S 51°16'21"W |  |  |
| 6V | 30°01'23"S 51°13'16"W |  |  |
| 7V | 30°01'46"S 51°13'55"W |  |  |
| 1J | 30°01'14"S 51°12'53"W |  |  |
| 2J | 30°01'25"S 51°13'11"W |  |  |
| 1N | 30°00'33"S 51°12'22"W |  |  |
| 2N | 30°00'37"S 51°12'10"W |  |  |
| 3N | 30°00'45"S 51°11'47"W |  |  |
| 9N | 30°00'21"S 51°11'30"W |  |  |
| 10N | 30°01'39"S 51°13'43"W |  |  |
| 11N | 30°01'33"S 51°13'29"W |  |  |

Table S2 presents the geographical coordinates of samples collected in campaigns 2 to 7 (22 locations in total).

Supplementary Table 2. Geographical coordinates of Campaign 2-7 collection sites.

| **Samples points** | **Coordinates** |
| --- | --- |
| P1 | 30°12'44"S 51°11'03"W |
| 2V | 30°00'52"S 51°15'32"W |
| 3V | 29°58'22"S 51°17'12"W |
| 4V | 29°59'31"S 51°16'21"W |
| P6 | 30°09'03"S 51°13'31"W |
| P7 | 30°08'36"S 51°13'38"W |
| P10 | 30°07'17"S 51°15'41"W |
| P13 | 30°06'19"S 51°15'44"W |
| P16 | 30°04'48"S 51°14'59"W |
| P26 | 30°02'38"S 51°14'10"W |
| P29 | 30°02'50"S 51°13'59"W |
| P30 | 30°03'04"S 51°14'02"W |
| P31 | 30°03'15"S 51°14'01"W |
| P32 | 30°03'38"S 51°14'13"W |
| P38 | 30°01'56"S 51°14'29"W |
| P39 | 30°01'45"S 51°14'12"W |
| P42 | 30°01'18"S 51°13'20"W |
| P43 | 30°00'38"S 51°12'48"W |
| P44 | 29°59'52"S 51°12'29"W |
| LAMI | 30°14'34"S 51°04'42"W |
| ITA | 30°16'58"S 51°01'18"W |

Table S3 lists the primer and probe sequences used for RT-qPCR detection of waterborne viruses. Targets included the 5’-UTR region of Hepatitis A virus (HAV), the ORF3 region of Hepatitis E virus (HEV), and the ORF1-2 junction region of Norovirus genogroups I and II (NoV I and NoV II).

Supplementary Table 3. - Primer sequences, concentrations and annealing cycling used for viral detection.

| **Target** | **Primer** | **Sequence** | **Annealing** | **Primers/**  **Probe [nM]** | **Reference** |
| --- | --- | --- | --- | --- | --- |
| HAV 5'-UTR | HAV-F | 5’- GGT AGG CTA CGG GTG AAA C -3’ | 55 °C for 45 s | 250 nM/ 150 nM | Jothikumar, 2005^1^ |
|  | HAV-R | 5’- AAC AAC TCA CCA ATA TCC GC -3 ’ |  |  |  |
|  | HAV-Probe | 5’- FAM CTT AGG CTA ATA CTT CTA TGA AGA GAT GC BHQ -3’ |  |  |  |
| HEV ORF3 | HEV-F | 5’-GGTGGTTTCTGGGGTGAC-3’ | 55 °C for 30 s | 250 nM/ 150 nM | Jothikumar, 2006^2^ |
|  | HEV-R | 5’-AGGGGTTGGTTGGATGAA-3’ |  |  |  |
|  | HEV-Probe | 5’- FAM TGATTCTCAGCCCTTCGC BHQ -3’ |  |  |  |
| NoV GI ORF1-2 | NoV GI-F | 5’-CGYTGGATGCGNTTYCATGA-3’ | 56 °C for 60 s | 320 nM/ 160 nM | Kageyama, 2003^3^ |
|  | NoV GI-R | 5’-CTTAGACGCCATCATCATTYAC-3’ |  |  |  |
|  | NoV GI-Probe | 5’-FAM-AGATYGCGATCYCCTGTCCA-TAMRA-3’ |  |  |  |
| NoV GII ORF1-2 | NoV GII-F | 5’-CARGARBCNATGTTYAGRTGGATGAG-3’ | 56 °C for 60 s | 320 nM/ 160 nM | Kageyama, 2003^3^ |
|  | NoV GII-R | 5’-TCGACGCCATCTTCATTCACA-3’ |  |  |  |
|  | NoV GII-Probe | 5’-HEX-TGGGAGGGCGATCGCAATCT-TAMRA-3’ |  |  |  |
| SARS-CoV-2 N1 | N1 PF | 5’- GAC CCC AAA ATC AGC GAA AT -3’ | 55 °C for 30 s | 300 nM/ 150 nM | CDC, 2020^4^ |
|  | N1 PR | 5’- TCT GGT TAC TGC CAG TTG AAT CTG -3’ |  |  |  |
|  | N1 Probe | 5’- FAM ACC CCG TAC GTT TGG TGG ACC BHQ -3’ |  |  |  |

Figure S1 illustrates the RT-qPCR standard curve for absolute quantification of the SARS-CoV-2 N1 genomic target, generated from serial dilutions of a ddPCR-quantified control. The linear regression yielded an equation of y = -3.295x + 33.088, with an amplification efficiency of 101.1% and a coefficient of determination (R^2^) of 0.994. The calibration plot and regression parameters were generated using the QuantStudio™ Design & Analysis Software (v1.5.2, Thermo Fisher Scientific).

Supplementary Figure 1 - RT-qPCR standard calibration curve, linear regression parameters, and amplification efficiency for SARS-CoV-2 (N1 target) quantification.

Results and Discussion

Table S4 shows the RT-qPCR results of genomic viral screening for each sample in the seven different sampling campaigns, displayed in cycle threshold values. Dots (•) represent not detected targets and hyphens (-) represent samples that were not collected in that campaign.

Supplementary Table 4. - Cycle threshold values for each virus in the different campaigns.

| **SAMPLE POOLS** | CAMPAIGN 1 | | | | | **SAMPLE** | CAMPAIGN 2 | | | | | CAMPAIGN 3 | | | | | CAMPAIGN 4 | | | | | CAMPAIGN 5 | | | | | CAMPAIGN 6 | | | | | CAMPAIGN 7 | | | | |
| --- | --- | --- | --- | --- | --- | --- | --- | --- | --- | --- | --- | --- | --- | --- | --- | --- | --- | --- | --- | --- | --- | --- | --- | --- | --- | --- | --- | --- | --- | --- | --- | --- | --- | --- | --- | --- |
|  | 8.May.24 | | | | |  | 21.May.24 | | | | | 4.jun.24 | | | | | 28.jun.24 | | | | | 28.Aug.24 | | | | | 29.nov.25 | | | | | 28.Apr.25 | | | | |
|  | SARS | HAV | HEV | NoV GI | NoV GII |  | SARS | HAV | HEV | NoV GI | NoV GII | SARS | HAV | HEV | NoV GI | NoV GII | SARS | HAV | HEV | NoV GI | NoV GII | SARS | HAV | HEV | NoV GI | NoV GII | SARS | HAV | HEV | NoV GI | NoV GII | SARS | HAV | HEV | NoV GI | NoV GII |
| 1RP | • | • | • | • | • | P1 | • | • | • | • | 30,34 | • | • | • | • | • | • | • | • | • | 32,92 | • | • | • | • | • | • | • | • | 38,24 | • | 33,5 | • | • | • | • |
| 3P | • | • | • | • | • | 2V | • | • | • | • | • | • | • | • | • | • | 37,44 | • | • | • | • | • | • | • | • | • | • | • | • | • | • | • | • | • | • | • |
| 4P | • | • | • | • | • | 3V | • | • | • | • | • | • | • | • | • | • | • | 37,54 | • | • | • | • | • | • | • | • | 37,09 | • | • | • | • | • | • | • | • | • |
| 5P | • | • | • | • | • | 4V | • | • | • | • | • | 37,46 | • | • | • | • | • | • | • | • | • | 36,77 | • | • | • | • | 36,78 | • | • | • | • | • | • | • | • | • |
| 6P | • | • | • | • | • | P6 | • | • | • | • | • | • | • | • | • | • | • | • | • | • | • | 36,57 | • | • | • | • | 36,71 | • | • | • | • | • | • | • | • | • |
| 8P | • | • | • | • | • | P7 | • | • | • | • | • | • | • | • | • | • | • | • | • | • | • | • | • | • | • | • | • | • | • | • | • | 32,28 | • | • | 36,22 | • |
| 9P | • | • | 38,21 | • | • | P10 | • | • | • | • | • | 36,59 | • | • | • | • | • | • | • | • | • | • | 37,15 | • | • | • | • | • | • | • | 35,39 | 35,26 | 39,17 | • | 37,13 | • |
| 10P | • | • | • | • | • | P13 | • | • | • | • | • | • | • | • | • | • | • | • | • | • | • | • | • | • | • | • | • | • | • | • | • | 26,83 | • | • | • | • |
| 11P | • | • | • | • | • | P14 | 37,2 | • | • | • | • | • | • | • | • | • | • | • | • | • | • | • | • | • | • | • | • | 37,31 | • | • | • | • | • | • | • | • |
| 13P | • | 37,71 | • | • | • | P16 | • | • | • | • | • | • | • | • | • | • | • | • | • | • | • | • | • | • | • | • | • | 38,27 | • | • | • | 38,81 | • | • | • | • |
| 14P | 35,03 | • | • | • | 37,4 | P26 | • | • | • | • | • | 36,53 | • | • | • | • | 38,82 | • | • | • | 34,4 | • | • | • | • | • | • | • | • | • | • | 26,85 | • | • | • | • |
| 15P | • | 38,36 | • | • | • | P29 | • | • | • | • | • | 33,95 | • | • | • | • | • | • | • | • | 37,31 | • | • | • | • | • | • | • | • | • | • | • | 35,4 | • | 36,04 | • |
| 16P | • | • | • | • | • | P30 | • | • | • | • | 30,76 | 35,23 | 35,92 | • | • | • | • | 35,79 | • | • | • | • | • | • | • | • | • | • | • | • | • | 27,07 | 38,09 | • | • | • |
| 17P | • | • | • | • | • | P31 | 36,86 | • | • | • | • | • | • | • | • | • | • | • | • | • | • | • | • | • | • | 36,19 | • | • | • | 37,91 | • | • | 36,83 | • | • | • |
| 18P | 34,24 | 37,43 | • | • | • | P32 | • | • | • | • | 38,6 | • | • | • | • | • | • | • | • | • | • | • | • | • | • | 38,5 | • | 36,25 | • | • | • | 27,26 | 36,07 | • | • | • |
| 19P | 36,73 | • | • | 22,9 | • | P38 | • | • | • | • | • | • | • | • | • | • | • | • | • | • | • | 37,62 | • | • | • | • | • | • | • | • | • | • | 35,91 | • | • | • |
| 20RP | • | • | • | • | • | P39 | • | • | • | • | • | • | • | • | • | • | • | • | • | • | • | • | • | • | • | • | 36,88 | • | • | • | • | 29,84 | • | • | • | • |
|  |  |  |  |  |  | P42 | • | • | • | • | • | • | 36,87 | • | • | • | • | • | • | • | 29,46 | 37,04 | • | • | • | • | • | • | • | • | • | 30,61 | 35,61 | • | • | • |
|  |  |  |  |  |  | P43 | • | • | • | • | • | • | • | • | • | • | • | 37,56 | • | • | • | • | • | • | • | • | • | 36,28 | • | • | • | 25,77 | • | • | • | • |
|  |  |  |  |  |  | P44 | • | • | • | • | • | • | • | • | • | • | • | • | • | • | • | • | • | • | • | • | • | • | • | • | • | 30,35 | • | • | • | • |
|  |  |  |  |  |  | LAMI | - | - | - | - | - | - | - | - | - | - | • | • | • | • | • | • | • | • | • | • | - | - | - | - | - | 26,01 | 37,95 | • | • | • |
|  |  |  |  |  |  | ITA | - | - | - | - | - | - | - | - | - | - | • | • | • | • | • | 39,89 | • | • | • | • | - | - | - | - | - | 29,6 | 37,29 | • | • | • |

- represents not detected samples; - represents site not collected for that campaign.
